# Cognitive performance and capacity to return home following out-of-hospital cardiac arrest

**DOI:** 10.1186/cc12243

**Published:** 2013-03-19

**Authors:** J Petrie, C Lockie, S Brett, R Stümpfle

**Affiliations:** 1 Imperial College Healthcare NHS Trust, London, UK

## Introduction

Before the introduction of primary percutaneous coronary intervention (PCI) and therapeutic hypothermia (TH) to out-of-hospital cardiac arrest (OOHCA) management, survival to hospital discharge with intact neurological function was poor [1,2]. We aimed to quantify the survival and degree of neurological impairment in OOHCA patients admitted to our ICU since the adoption of post-OOHCA bundles.

## Methods

Sixty-nine consecutive OOHCA patients admitted to the ICU at Hammersmith Hospital from 1 January 2011 to 30 June 2012 were identified and reviewed from hospital databases. Cognitive status was scored using Cerebral Performance Category (CPC); 1 to 2 normal-mild and 3 to 4 moderate-severe neurological impairment. Scores were determined from ICU summaries, occupational and physiotherapy reports. Hospital discharge outcomes were determined from hospital databases.

## Results

TH was initiated in 93% (64/69) of OOHCA patients and 87% (40/46) with ischaemic cardiac aetiology underwent PCI. ICU survival was 58% (40/69); 65% (26/40) scoring CPC 1 to 2 and 35% (14/40) CPC 3 to 4 at ICU discharge. Two patients with CPC 2 improved to CPC 1 during their hospital stay. All patients with CPC 1 to 2 survived to hospital discharge; two required general rehabilitation before returning home. Only 43% (6/14) of CPC 3 to 4 patients survived to hospital discharge; none returned home. Two went into hospice care, one was repatriated to another hospital and three went to neuro-rehab. No CPC 3 to 4 patients improved CPC scores after ICU discharge. Overall hospital survival was 46%. See Table 1.

**Table 1:** Discharge and neurological status of OOHCA patients

		Discharge		Hospital discharge destination		
		
						
**Score**	**ICU**	**Hospital**	**Home**	**Neuro-rehab**	**Repatriation**	**Hospice**
CPC 1	20	22	19	0	3	0
CPC 2	6	4	4	0	0	0
CPC 3	4	3	0	3	0	0
CPC 4	10	3	0	0	1	2
Total	40	32	23	3	4	2

## Conclusion

OOHCA patients admitted to our ICU had a 46% chance of surviving to hospital discharge. Most patients left hospital with good neurological status (CPC 1 to 2); moderate-severe neurological disability (CPC 3 to 4) was seen in 19%, greater than previously reported [1]. A higher proportion (35%) of patients discharged from the ICU had moderate-severe neurological disability; most subsequently died in hospital (62%). These figures may represent better ICU outcomes subsequent to adoption of OOHCA bundles but suggest further work is required in neuro-disabled survivors.
